# The Roles of Kisspeptin System in the Reproductive Physiology of Fish With Special Reference to Chub Mackerel Studies as Main Axis

**DOI:** 10.3389/fendo.2018.00147

**Published:** 2018-04-04

**Authors:** Hirofumi Ohga, Sethu Selvaraj, Michiya Matsuyama

**Affiliations:** Laboratory of Marine Biology, Faculty of Agriculture, Kyushu University, Fukuoka, Japan

**Keywords:** kisspeptin, puberty, brain–pituitary–gonad axis, marine teleost, perciform, aquaculture

## Abstract

Kisspeptin, a novel neuropeptide product of the *Kiss1* gene, activates the G protein-coupled membrane receptor G protein-coupled receptor 54 (now termed Kiss1r). Over the last 15 years, the importance of the kisspeptin system has been the subject of much debate in the mammalian research field. At the heart of the debate is whether kisspeptin is an absolute upstream regulator of gonadotropin-releasing hormone secretion, as it has been proposed to be the master molecule in reproductive events and plays a special role not only during puberty but also in adulthood. The teleostean kisspeptin system was first documented in 2004. Although there have been a number of kisspeptin studies in various fish species, the role of kisspeptin in reproduction remains a subject of controversy and has not been widely recognized. There is an extensive literature on the physiological and endocrinological bases of gametogenesis in fish, largely derived from studying small, model fish species, and reports on non-model species are limited. The reason for this discrepancy is the technical difficulty inherent in developing rigorous experimental systems in many farmed fish species. We have already established methods for the full life-cycle breeding of a commercially important marine fish, the chub mackerel (cm), and are interested in understanding the reproductive function of kisspeptins from various perspectives. Based on a series of experiments clarifying the role of the brain–pituitary–gonad axis in modulating reproduction in cm, we theorize that the kisspeptin system plays an important role in the reproduction of this scombroid species. In this review article, we provide an overview of kisspeptin studies in cm, which substantially aids in elucidating the role of kisspeptins in fish reproduction.

## Introduction

Kisspeptin is an RFamide peptide product of the *Kiss1* gene and the natural ligand of the G protein-coupled receptor 54 (GPR54), now named Kiss1r (1, 2). Mature kisspeptins in mammals are cleaved into endogenous fragments: Kp54, Kp16, Kp14, Kp13, and Kp10 (3). The C-terminus decapeptide Kp10 (Kiss-10) region is the minimum active site and is highly conserved across vertebrates. Mutations in the *Kiss1R* gene are correlated with an absence of puberty onset and hypogonadotropic hypogonadism in humans (4, 5). These abnormalities are due to the disruption of the hierarchical reproductive network, especially the kisspeptin–gonadotropin-releasing hormone (Gnrh)–luteinizing hormone (Lh) pathway. Kisspeptin fibers have been observed in the vicinity of Gnrh neuron cell bodies, and a large population of Gnrh neurons expresses *Kiss1r* mRNA, clearly indicating that kisspeptin neurons directly signal Gnrh neurons (6–8). Indeed, administration of Kp10 was found to elicit a robust increase in the circulating levels of Gnrh (9). Several studies in mammals have strongly demonstrated the absolute necessity of kisspeptin signaling for puberty onset and ovulation through the regulation of Gnrh secretion.

In 2004, the isolation of the complementary DNA (cDNA) of a piscine ortholog of the kisspeptin receptor (*kissr2*) in Nile tilapia (*Oreochromis niloticus*) was the first evidence for the existence of a kisspeptin system in fish (10). In mammals, only one gene (*Kiss1*) coding for the ligand and one for the receptor (*Kiss1r*) are present. Teleost fishes, known to have undergone a third genome duplication event, have two paralogous kisspeptin genes (*kiss1* and *kiss2*), and four genes encoding kisspeptin receptors have been reported, although most fish have only two receptors, *kissr2* and *kissr3* (also known as *gpr54-2b* and *gpr54-1b*) (11, 12).

At least two molecular forms of Gnrh are present in the brains of all vertebrate species, with some teleosts expressing three different forms (i.e., Gnrh1, Gnrh2, and Gnrh3) (13). Gnrh1 is considered the major hypophysiotropic hormone controlling the synthesis and release of Lh in all vertebrates. By contrast, Gnrh3 is a teleost-specific form that is expressed in neuronal populations in the olfactory bulb, the terminal nerve ganglion region, and the pre-optic area (POA). Gnrh3 axonal fibers project into different brain regions, suggesting a role in neuromodulation. In fish expressing two Gnrh forms, such as the Salmonidae and Cyprinidae, Gnrh3 not only functions as a neuromodulator but also regulates the secretion of pituitary gonadotropins (14, 15).

The major underlying question is whether fish kisspeptin is an important regulator of the reproductive brain–pituitary–gonad (BPG) axis. Several studies strongly suggest that, similar to its mammalian counterpart, the fish kisspeptin ortholog is a potent activator of the reproductive axis. Gnrh1 neurons express *kissr2* mRNA in cichlid fish (e.g., *O. niloticus* and *Astatotilapia burtoni*) (10, 16), and striped bass (*Morone saxatilis*) (17) is a notable example. In addition, several *in vivo* studies have shown that the injection of Kiss2 peptide promotes the secretion of Gnrh and gonadotropins (18, 19). Furthermore, kisspeptin antagonists were found to inhibit sperm production in striped bass (20). However, in the zebrafish (*Danio rerio*), *kiss-* or *kissr-*knockout mutants exhibited normal gonadal maturation, indicating that kisspeptin signaling is not indispensable for reproduction in this species (21). A few studies have indicated that Gnrh neurons do not express kisspeptin receptors in medaka (*Oryzias latipes*) (22) or European sea bass (*Dicentrarchus labrax*) (23). Hence, the true role of kisspeptin in fish reproduction remains open to debate.

The chub mackerel (cm) (*Scomber japonicus*) is a small marine pelagic fish, which belongs to the order Perciformes and the family Scombridae. This species is one of the top 10 principal food fish contributing to global capture fisheries production. In addition, cm is a suitable experimental model fish for reproductive endocrinological research in Perciformes, which is the most evolved and largest teleost fish group and includes many target aquaculture species. Our team recently developed standardized methods to support the full life cycle of this species in land-based small-scale aquaculture. This system aids in obtaining a series of captive cm samples throughout the entire reproductive cycle for the analysis of key hormones acting in the BPG axis and enables breeding experiments. Using these fish sampling facilities, our group has isolated the key molecular elements of the cm BPG axis in reproduction, namely Gnrhs (Gnrh1, 2, and 3) (24, 25), the Gnrh receptor (GnrhR1) (26), gonadotropic hormones (Gths) (27–30), and Gth receptors (31). Furthermore, steroid hormones involved in vitellogenesis and oocyte maturation have been demonstrated in this species (32). There is an extensive literature on the physiological and endocrinological bases of gametogenesis in fish; this literature is largely derived from small, model fish species, such as zebrafish and medaka. However, in many cases, data on the mechanisms of reproductive regulation in teleosts vary greatly among species. This may be due to the considerable length of their evolutionary process and the diversity of species, reproductive patterns, and habitats. To clarify the general mechanisms underlying the reproductive physiology of fish, studies in non-model species may be informative.

In recent years, we have focused on inducing the potency of kisspeptin peptides in gonadal development. Of particular interest is the period of pubertal transition and gonadal recrudescence, which is important for the establishment of an efficient aquaculture of any fish species. The aim of this article is to review our previous 11 papers on the kisspeptin system in cm reproduction. At the same time, we strongly adhere to a comparative approach involving other model and non-model teleost fish species to provide a comprehensive summary of the knowledge on fish kisspeptins to date.

## Characteristics of Kisspeptins and Their Receptors

### Ligands

The cm possesses two kisspeptin genes, *kiss1* and *kiss2* (33). These sequences were submitted to GenBank as follows: *kiss1*, GU731672 and *kiss2*, GU731673. *cmkiss1* and *cmkiss2* cDNAs encode 105 and 123 amino acids, respectively, and display very low sequence similarity (18%).

Kisspeptin coding sequences have been isolated in many fish species. All reported teleost species possess the *kiss2* gene, whereas the genomes of puffer fish and sticklebacks (*Gasterosteus aculeatus*) lack the *kiss1* gene and contain only the *kiss2* gene (18).

The C-terminus decapeptide Kp10 region is highly conserved within mammalian and non-mammalian vertebrates. In an initial study of fish kisspeptin, the deduced sequences for Kiss1-10 and Kiss2-10 were assumed to be the minimum functional core peptides. The modified fish KP44 peptide, which lacks the C-terminus KP10 region, has no bioactivity, suggesting that the KP10 region is essential for receptor binding, as is the case with mammalian kisspeptin (34). However, in teleost fish, the Kiss1 precursor contains a conserved dibasic 5-amino acid site upstream of the KP10 region, indicating that it produces a mature pentadecapeptide (Kiss1-15), which should have pyroglutamate at the N-terminus because the residue at the N-terminal end of Kiss1-15 is glutamine in all reported fish species (35). Similarly, a conserved arginine residue at position 13 is present in all available Kiss2 sequences, indicating the presence of a putative cleavage site that produces a mature dodecapeptide (Kiss2-12) (35). Both the Kiss1-15 and Kiss2-12 regions are highly conserved across teleost fishes.

The cmKiss1-15 (QDMSSYNFNSFGLRY-NH_2_) and cmKiss2-12 (SNFNFNPFGLRF-NH_2_) peptides showed the highest potency for the activation of cognate receptors, stronger than their corresponding KP10 peptides in cm (36). The same results were reported in zebrafish (35) and European sea bass (37). These results suggest that amino acid sequences other than the KP10 region are also functionally important, perhaps due to factors such as the structure, hydrophobicity, or electric charge of fish kisspeptins. Choosing the right peptide form to test is therefore important for clarifying the bioactivity of fish kisspeptins.

The cmKiss1 precursor contains a putative cleavage site located seven amino acids upstream of the core sequence (33), indicating that it produces a mature hexadecapeptide (cmKiss1-16: HQDMSSYNFNSFGLRY-NH_2_). This Kiss1-16 showed higher sensitivity for receptor activation than Kiss1-15 (38). Another example, a mature Kiss2 tridecapeptide (Kiss2-13) was isolated in masu salmon (*Oncorhynchus masou masou* and *Oncorhynchus nerka*) (39). This may also reflect differences in the basic site of the precursor protein.

### Receptors

Chub mackerel possess two receptor subtypes (*kissr2* and *kissr3*), which have already been submitted to GenBank as *kissr2* (*gpr54-2*): JX982323 and *kissr3* (*gpr54-1*): JX982322 (36). The *kissr2* and *kissr3* cDNAs have open reading frames of 1,137 bp (378 amino acids) and 1,110 bp (369 amino acids), respectively. The amino acid sequences of the receptors share only 52% identity; however, high sequence identity was found between the transmembrane domains (73%). cmKissR2 exhibited >80% identity with all other teleosts and >93% identity with other Perciform fish KissR2 sequences. cmKissR3 showed high similarity to European sea bass (88%), followed by striped bass (87%); however, slightly lower homology with goldfish (65%) and zebrafish (62%) KissR3 sequences was observed.

Since its first discover in 2004, kisspeptin receptors have been cloned and sequenced in more than 50 different teleost fish species. Almost all these fish possess the piscine ortholog *kissr2*, whereas few species possess *kissr2* and *kissr3* sequences in their genomes. There are a few exceptions; for example, the European eel (*Anguilla anguilla*), coelacanth (*Latimeria chalumnae*), and spotted gar (*Lepisosteus oculatus*) possess three types of receptors (*kissr1, kissr2*, and *kissr3*), and coelacanth and the spotted gar further possess *kissr4* genes, as detailed in the phylogenetic tree (11, 40). It is important to note that the nomenclature of fish kisspeptin receptors has not yet been established, and different research teams use different abbreviations. In this article, the receptor names are adopted from syntenic studies by Pasquier et al. (11, 12). Elsewhere, *kissr2* is sometimes referred to as *kiss2r* or *gpr54-2*. Furthermore, other designations of *kissr3* include *kissr1, kiss1r*, and *gpr54-1*, because, in many cases, this receptor showed high sensitivity for the Kiss1 peptide. Our previous kisspeptin papers referred to the kisspeptin receptors as *kissr1* and *kissr2*.

### Ligand Sensitivity

**Figure 1:**
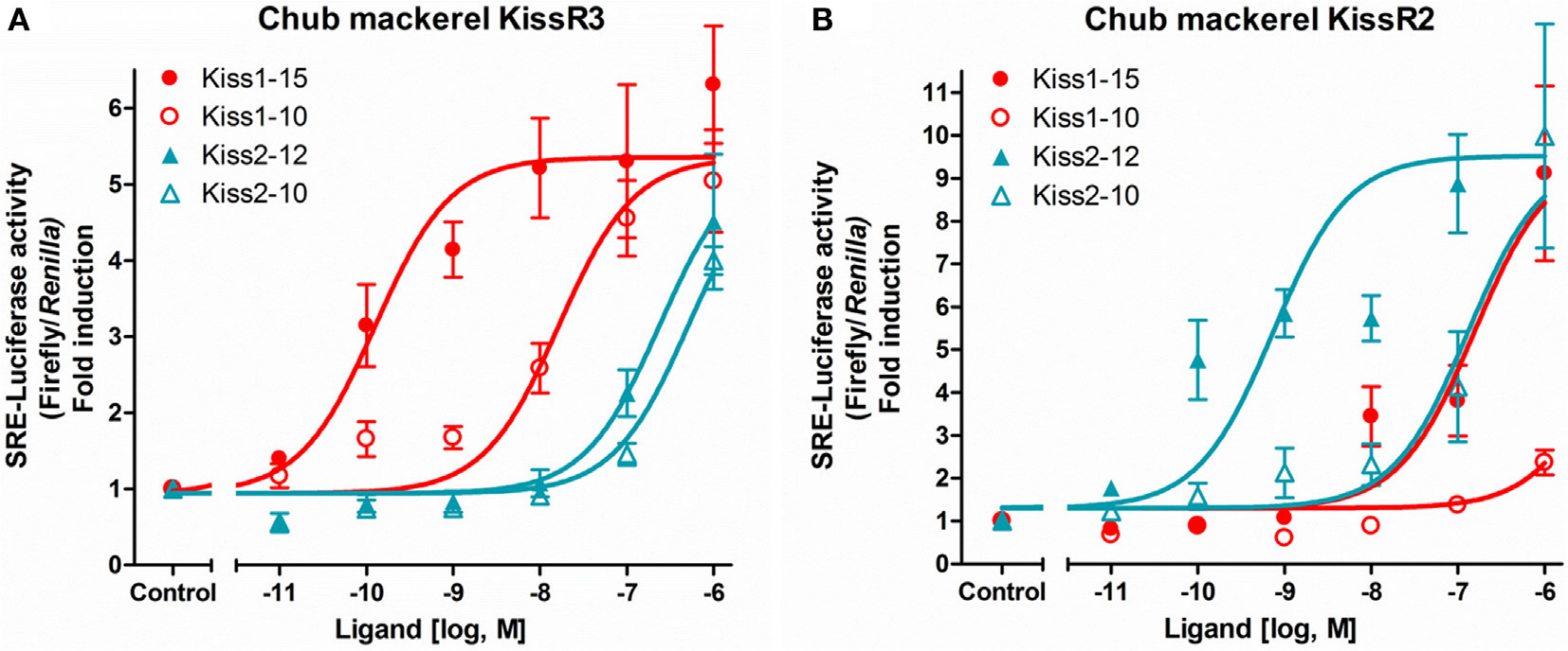
Ligand selectivity of the chub mackerel (cm) kisspeptin receptors. KissR3 **(A)** and KissR2 **(B)**, each together with SRE-Luc. Transfected cells were treated with graded concentration of each peptides. The data are expressed as the ratio of changes in firefly luciferase activity over the control *Renilla* luciferase activity. Each point was determined in quadruplicate and is given as a mean ± SEM. Modified from Ref. (36), by permission of Elsevier.

The ligand sensitivities of the two subtypes of cm kisspeptin receptors were examined by reporter gene assays using mammalian cell lines. The results revealed that cm kisspeptin receptor signals are preferentially transduced *via* the protein kinase C (PKC)/mitogen activated protein kinases rather than by the protein kinase A (PKA) pathway (36). Synthetic cm Kiss1-15 (or Kiss1-16) and Kiss2-12 peptides showed the highest potency for the activation of KissR3 and KissR2, respectively (Figures 1A,B) (36, 38). Thus, we concluded that KissR3 and KissR2 are the intrinsic receptors for the Kiss1 and Kiss2 peptides, respectively, and signals are mainly transduced *via* the PKC pathway in this species (36).

Ligand sensitivity has been analyzed in duplicated kisspeptin systems in zebrafish (35, 41), goldfish (*Carassius auratus*) (42), Southern bluefin tuna (*Thunnus maccoyii*) (43), yellowtail kingfish (*Seriola lalandi*) (43), medaka (22), European sea bass (37), and striped bass (20) and, in one species, the orange-spotted grouper (*Epinephelus coioides*), with only the Kiss2/KissR2 pair (44). In all studies, intracellular signals were preferentially transduced *via* the PKC pathway; however, the PKA pathway was also activated by ligand stimulation in goldfish (42), medaka (22), and European sea bass (37). In many cases, KissR2 and KissR3 showed high sensitivity for the Kiss2 and Kiss1 peptides, respectively. Conversely, in goldfish, Kiss1-10 enhanced KissR2 activation, and Kiss2-10 exhibited a higher preference for KissR3 (42). It should also be noted that KissR2 showed equal sensitivity to both Kiss1 and Kiss2 peptides in the zebrafish (35), Southern bluefin tuna, and yellowtail kingfish (43); KissR3 showed the same binding potency for Kiss1 and Kiss2 peptides in striped bass (20). The high species specificity and the complexity of the ligand sensitivity or signaling pathways in duplicated kisspeptin systems in fish is noteworthy.

## Bioactivity

### Acute Effects

**Figure 2:**
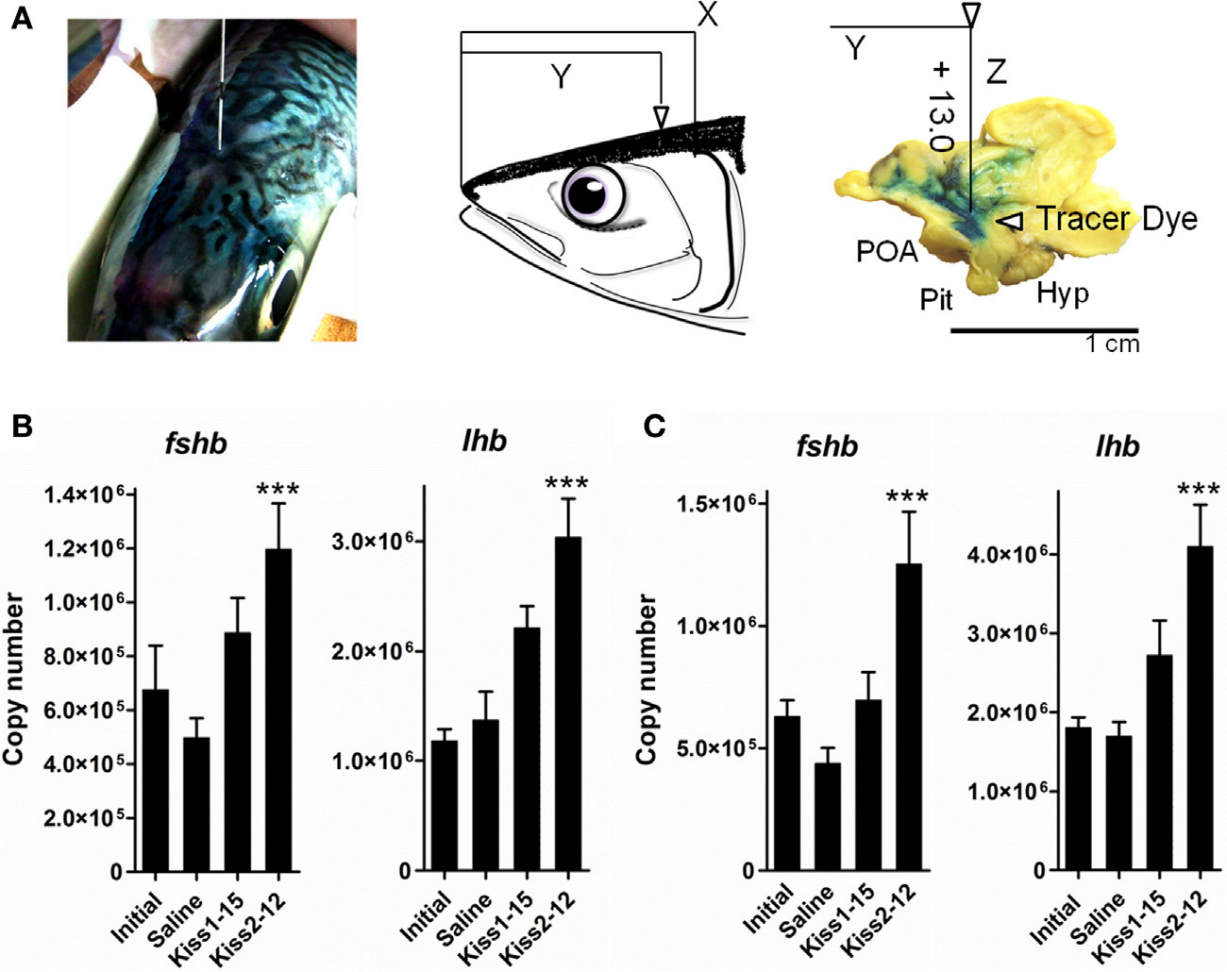
Schematic illustration of coordinates for injection into the intracerebroventricular (i.c.v.) region of chub mackerel **(A)**. We determined that if head length (rostral end to upper end of gill) was assumed to be *X* and length of the rostral end to immediately above the third ventricle was assumed to be *Y*, the ratio of *X* to *Y* converged from 1.4 to 1.0. Tested peptide or PBS with blue dye was administered into the third ventricle to a depth of 13 mm below the tissue surface. Abbreviations: Hyp, hypothalamus; Pit, pituitary; POA, pre-optic area. **(B,C)** Analysis of the effect of i.c.v. administration of Kiss1-15 and Kiss2-12 peptides on *fshb* and *lhb* mRNA levels in the pituitary at 12 h post-administration, each together in adult male **(B)** or female **(C)** immature subjects. Transcription levels are the mean ± SEM of 9–16 independent determinations. ***P < 0.001, one-way ANOVA followed by a Tukey’s multiple comparison test. Modified from Ref. (45), by permission of Elsevier.

We evaluated the biological potency of kisspeptin peptides to induce transcriptional changes in *gnrh1*, follicle-stimulating hormone *(fsh)b*, and *lhb* in gonadal recrudescent cm. Synthetic Kiss1-15 and Kiss2-12 were administered at a dose of 100 ng into the intracerebroventricular (i.c.v.) region, and brains were sampled at 6 and 12 h post-administration (Figure 2A). In both sexes, the transcription levels were quantified using quantitative real-time PCR (qRT-PCR). i.c.v. administration of Kiss2-12 but not Kiss1-15 significantly elevated pituitary *fshb* and *lhb* transcripts at 12 h post-administration in comparison to saline-injected controls (Figures 2B,C) (45). In addition, in females, the transcription levels of POA *gnrh1* were downregulated by both kisspeptin peptides at 12 h post-administration (45). It is clear that central administration of kisspeptin peptides influenced Gnrh and gonadotropin synthesis, suggesting that kisspeptin peptides may play a central role in the regulation of the reproductive BPG axis in cm.

The third ventricle was chosen as the site of administration due to its proximity to the brain centers controlling reproductive activity; it is the most accurate way to study the role of centrally acting peptides (45). Thus, delivering exogenous kisspeptins to the central nervous system is important, and our study was the first to demonstrate the effects of kisspeptin peptides on brain *gnrh* mRNA expression. In the same manner, Espigares et al. evaluated the bioactivity of kisspeptin peptides in immature adult European sea bass. i.c.v. administration of Kiss2-12 stimulated Gnrh1 release into the pituitary and increased serum Fsh, Lh, and sex steroids until 72 h post-treatment (19). These results suggest that kisspeptin potently stimulates reproductive axis activity.

The biological effects of kisspeptin peptides through peripheral [intraperitoneal (i.p.) and intramuscular (i.m.)] administration have been studied in some fish species. In early to mid-pubertal fathead minnows (*Pimephales promelas*), i.p. administration of mammalian KP10 increased the expression of *gnrh3* in the brain after 10 h (46). In sexually mature female zebrafish, i.p. administration of Kiss2-10 upregulated *fshb* and *lhb* expression 12 h post-treatment (47). In goldfish, i.p. administration of Kiss1-10 stimulated Lh secretion until 6 h post-treatment (42). In sexually mature female orange-spotted grouper, i.p. administration of Kiss2-10 increased hypothalamic expression of *gnrh1* and pituitary *fshb* expression until 12 h post-treatment (44). Finally, in pubertal hybrid bass (*Morone* species), i.m. administration of Kiss2-12 upregulated plasma Lh levels until 24 h post-treatment and, during gonadal recrudescence, both Kiss1-15 and Kiss2-12 induced Lh secretion after 24 h (17). Taken together, these data conclusively demonstrate that kisspeptins induce Gnrh and gonadotropin release in many fish species.

### Chronic Treatment *In Vivo*

We evaluated the potency of Kiss1 and Kiss2 in inducing gonadal development in sexually immature prepubertal and gonadal recrudescent cm. In pubertal fish, synthetic Kiss1-15 or Kiss2-12 peptides were mixed with molten cocoa butter (slow-releasing medium), and peripheral injection was repeated three times at an interval of 2 weeks. In adult fish, the same peptides were administered subcutaneously using Alzet mini-osmotic pumps (Model 2006). In both studies, peptide treatments were continued for a period of 6 weeks.

**Figure 3:**
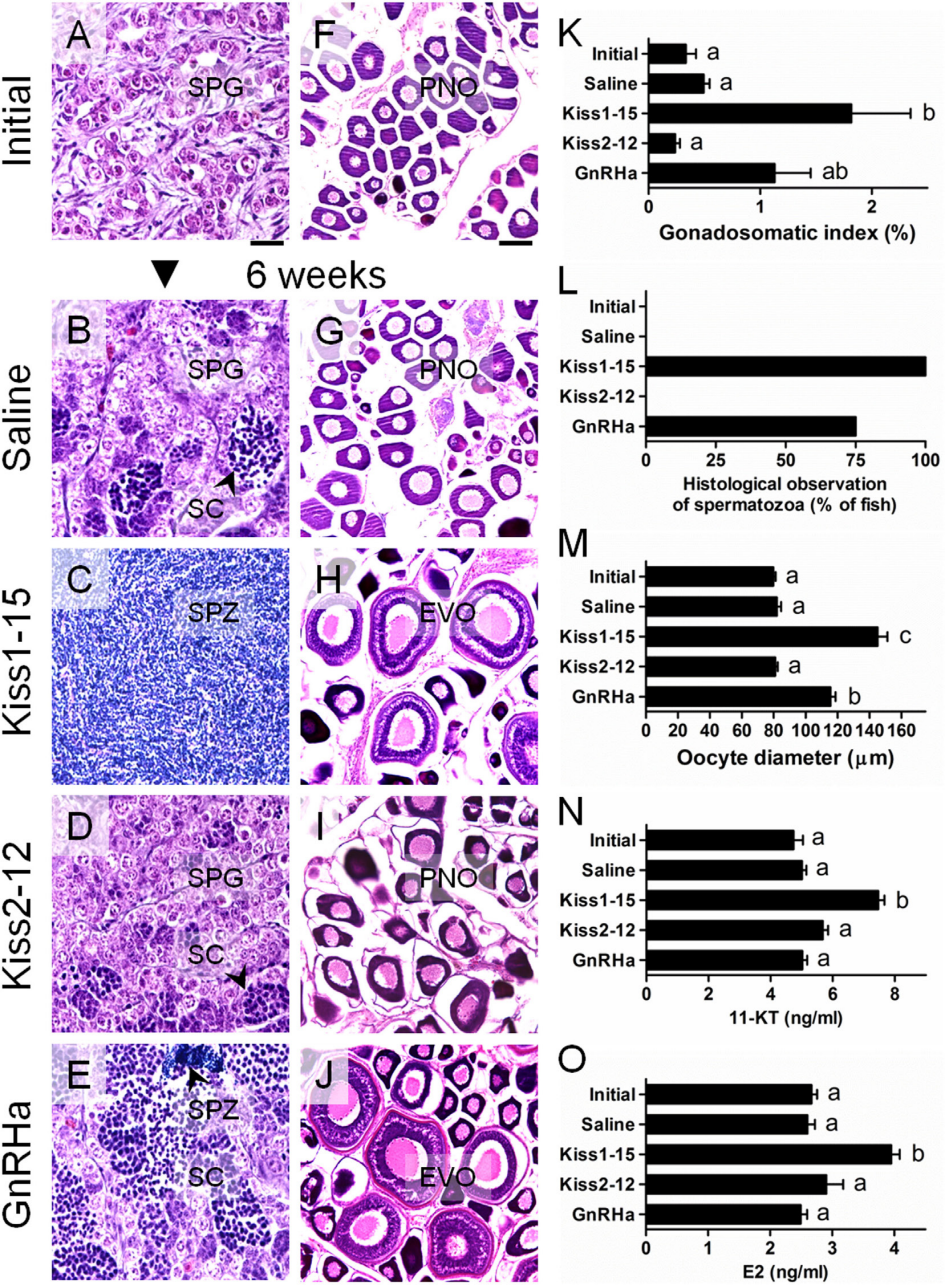
Changes in the gonadal histology of male **(A–E)** and female **(F–J)** chub mackerel in different treatments. Abbreviations; SPG, spermatogonia; SC, spermatocytes; SPZ, spermatozoa; PNO, perinucleolar oocyte; EVO, early vitellogenic oocyte. Scale bars = 100 µm. **(K)** Changes in the gonadosomatic index (GSI) of male fish. **(L)** Percentage of fish by treatment showing histological presence of SPZ in the testes. **(M)** Changes the mean oocyte diameter of fish at different treatments, respectively. Changes in serum 11-ketotestosterone (11-KT) in male **(N)** and estradiol-17b (E2) in female **(O)**, respectively. Values are means ± SEM (*n* = 4–6 for each treatment). Different letters above the bars represent significant differences between treatments (*p* < 0.05, one-way ANOVA followed by a Tukey’s multiple comparison test). GnRHa: GnRH analog (D-Ala^6^, des-Gly^10^)-LHRH ethylamide. Modified from Ref. (50), by permission of Zoological Society of Japan.

These results deserve explicit emphasis. In pubertal males, 66.7% of Kiss1-15-treated fish had spermatozoa (SPZ) in their testes, whereas no SPZ were observed in Kiss2-12- and saline-treated fish (48). In addition, the levels of sex steroids 11-ketotestosterone (11-KT) and estradiol-17β (E2) were significantly higher in Kiss1-15-treated fish (48). In pubertal females, Kiss1-15-treated fish displayed yolk vesicles in the growing oocytes, whereas Kiss2-12- and saline-treated fish did not. E2 levels were significantly higher in Kiss1-15-injected fish than in control fish (49). The effects on gonadal recrudescence were more critical. In recrudescent males, Kiss1-15-treated fish exhibited a significantly higher gonadosomatic index (GSI) than did all other treatment groups, and SPZ were present in the testes of Kiss1-15-treated fish at the end of the experiment (Figures 3A–E,K,L) (50). The GSI values of adult females did not differ among treatments; however, the mean oocyte diameters of Kiss1-15-treated fish, representing the early vitellogenic oocytes, were significantly higher than those of the saline- and Kiss2-12-treated fish (Figures 3F–J,M) (50). In both sexes, Kiss1-15-treated fish exhibited higher levels of circulating 11-KT and E2 (Figures 3N,O) (50). These results suggest that synthetic kisspeptin peptides can induce pubertal onset and gonadal recrudescence in cm.

There is ample evidence to indicate the importance of kisspeptin stimulation for gonadotropin secretion and gonadal development in fish. Beck et al. reported that subcutaneous biweekly administration of European sea bass Kiss1-10 but not Kiss2-10 for 7 weeks significantly increased the GSI and SPZ volume in prepubertal male white bass (*M*. *chrysops*) (51). Nocillado et al. utilized slow-release implants to chronically deliver synthetic kisspeptin to prepubertal male yellowtail kingfish and reported that fish treated with Kiss1-10 were 100% developed over 4 weeks and exhibited the most advanced stage of development, with testes containing mostly spermatids and SPZ (52). On the other hand, 8-week treatment with Kiss2-10 had a stronger stimulatory effect on testicular development during the non-breeding period (52). In male and female cinnamon clownfish (*Amphiprion melanopus*), treatment with human KP10 upregulated Gnrhs, Gths, Gth receptors, estrogen, and vitellogenin in the brain, pituitary, gonads, serum, and liver, respectively, and also promoted gonadal development over 6 weeks (53). In male European sea bass, Kiss2-12 treatment induced a significant increase in cumulative milt on days 3 and 7 after i.c.v. administration (19). Finally, in male and female Nile tilapia, cognate Kiss2-10 was administered i.p. twice weekly, and this increased the expression of *gnrh1, fshb* and *lhb* mRNA, and plasma levels of E2 and 11-KT (54). This study also showed accelerated testicular development after 4 weeks (with eight total administrations of kisspeptin). Overall, these studies suggest that chronic treatment with kisspeptin peptides modulates gonadotropin secretion and influences gonadal development in many fish species. However, the effects on ovarian development may be slower. For example, oocyte size increased, but there were no histological differences in prepubertal white bass after a 7-week period of Kiss1-10 or Kiss2-10 treatment (51), in prepubertal striped bass after 10 weeks of Kiss1-15 treatment (55), or in immature Nile tilapia after 4 weeks of Kiss2-10 treatment (54). The same trend was confirmed in our study using prepubertal female cm (49). Detailed and long-term research is needed to comprehensively examine both the basic science and commercial application of kisspeptin peptides.

## Gene Expression

### Expression in BPG Axis

Quantitative real-time PCR analysis revealed that kisspeptin system transcripts are distributed in different tissues of the BPG axis in adult male and female cm. The *kiss1* gene was expressed in the brain, pituitary, and gonad (33). The *kiss2* gene was expressed only in the brain (33). The *kissr2* gene was expressed in the brain, pituitary, and testes but not in the ovary (36). The *kissr3* gene was expressed in the brain and testes but not in the ovary (36).

### Early Developmental Stages

We analyzed expression changes in these genes during early development [0–30 days post-hatching (dph)] and during the period of gonadal sex differentiation (37–60 dph) in cm using qRT-PCR assays. During early development, the expression of *kiss1, kiss2*, and *kissr2* in the whole head did not vary significantly; however, *kissr3* expression decreased significantly at 20 dph compared with expression levels just after hatch (56). Interestingly, *kiss2, kissr2*, and *kissr3* were significantly elevated at the start of gonadal sex differentiation in both males and females (56). These results suggest the potential involvement of the kisspeptin system during early development and gonadal sex differentiation in the cm.

There is strong evidence to suggest that the early kisspeptin system modulates the proliferation of the neuronal network. In zebrafish, Zhao et al. showed that early treatment with both Kiss1 and Kiss2 stimulated the proliferation of trigeminal Gnrh3 neurons located in the peripheral nervous system, and Kiss1, but not Kiss2, stimulated the proliferation of the terminal nerve and hypothalamic populations of Gnrh3 neurons in the central nervous system (57). In the same species, qRT-PCR revealed detectable levels of *kiss1* and *kiss2* mRNA by 1 day post-fertilization (dpf), which increased throughout embryonic and larval development (57). Furthermore, there are several other examples of kisspeptin expression at early developmental stages. In medaka, the expression of *kiss1, kiss2*, and *kissr3* was observed immediately 1 h post-fertilization, and both *kiss1* and *kiss2* levels peaked at 1 dpf (58). In cobia (*Rachycentron canadum*), *kissr2* expression was detected at 1 dph and peaked at 2 and 3 dph (59). In black rockfish (*Sebastes schlegelii*), both *kiss1* and *kissr2* expression displayed an increasing trend during early development (60).

Expression changes during gonadal sex differentiation were also confirmed in some reports. In cobia, *kissr2* expression increased in sexually differentiated males but not in females (59). In fathead minnow, *kissr2* expression increased in both males and females immediately after gonadal sex differentiation (46). In pejerrey (*Odontesthes bonariensis*), *kiss2* levels increased at 4 weeks after hatching, which is when gonadal differentiation occurs in this species (40). Finally, in tongue sole (*Cynoglossus semilaevis*), *kiss2* and *kissr2* transcripts rapidly increased during the early gonadal differentiation period (61). It is likely that an early kisspeptin system is important in brain development and involved in modulating gonadal sex differentiation in teleost fishes.

### Pubertal Stage

**Figure 4:**
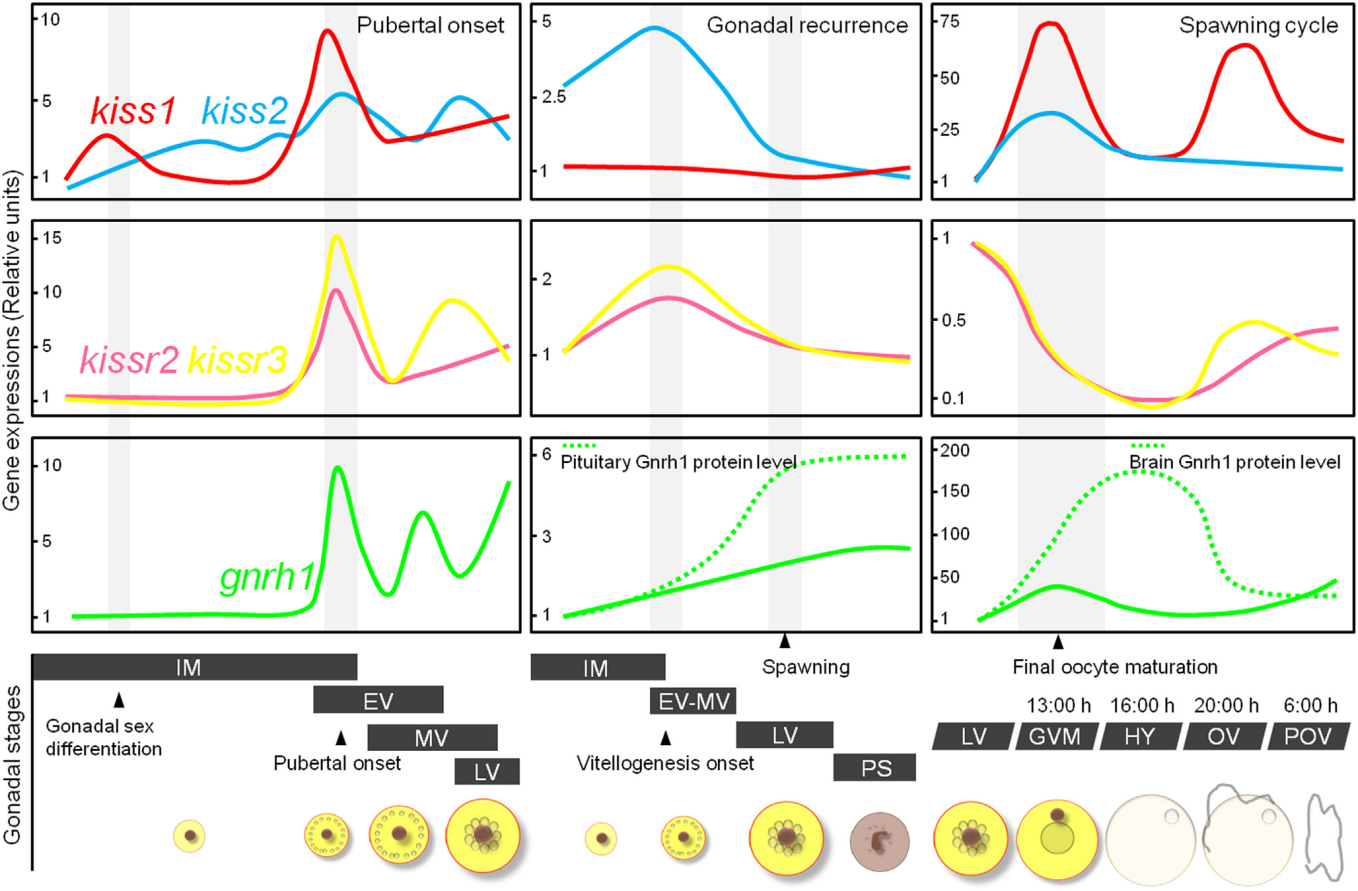
Schematic representation showing relative changes in *kiss1, kiss2, kissr2, kissr3*, and *gnrh1* gene levels in the brain of female chub mackerel during the puberty, gonadal recurrence, and spawning cycle. Abbreviations: IM, immature; EV, early vitellogenesis; MV, mid-vitellogenesis; LV, late vitellogenesis; GVM, germinal vesicle migration; HY, hydration; OV, ovulation; POV, post-ovulation.

We examined the temporal patterns of gene expression of the two kisspeptin subtypes and their receptors in the brain during the pubertal process in cm using qRT-PCR. In male fish, *kiss2* and *gnrh1* expression levels increased significantly just before the onset of meiosis in the testes (62). In female fish, *kiss1* and *kiss2* levels increased significantly, concomitant with increases in the *kissr2, kissr3*, and *gnrh1* levels, just before the onset of vitellogenesis in the oocytes (Figure 4) (62). Notably, upon pubertal onset, pituitary *gnrhr1, fshb*, and *lhb* began to gradually increase in both sexes (26, 63). These results suggest that, as is the case in mammals, the positive involvement of the kisspeptin system in the pubertal process in cm.

For the moment, let us closely examine kisspeptin gene expression at puberty. The levels of *kissr2* mRNA in the brain were correlated with pubertal development in the cobia (59), grey mullet (*Mugil cephalus*) (64), zebrafish (41), fathead minnow (46), Nile tilapia (65), Senegalese sole (*Solea senegalensis*) (66, 67), Atlantic halibut (*Hippoglossus hippoglossus*) (68), and Atlantic salmon (*Salmo salar*) (69). In the brains of Nile tilapia, *kissr2* was expressed in a significantly higher percentage of Gnrh neurons in mature males than in immature males (10). Ligand expression during puberty was reported in female zebrafish, and *kiss1, kiss2* and *gnrh3* all showed increased gene expression at the onset of puberty (47). Similarly, brain *kiss2* expression levels increased significantly in flat fish, Senegalese sole (67, 70) and Japanese flounder (71). In Atlantic cod (*Gadus morhua*), acute *kiss2* elevation was observed in maturing individuals (72). In red sea bream (*Pagrus major*), the number of *kiss2*-expressing neurons in the hypothalamus was greater during the first spawning season in both males and females compared with fish in the post-spawning periods (73). In female striped bass, *kiss1* and *kiss2* and their receptors were dramatically elevated in mature females compared with prepubertal levels (17). These results clearly demonstrate that kisspeptin gene expression and pubertal timing are highly correlated in many teleost fish species. These results support the involvement of the kisspeptin system in pubertal onset in fish reproduction.

### Annual Reproductive Stage

To elucidate the involvement of multiple kisspeptin systems in annual gonadal recurrence in cm, their relative gene expression profiles in the brain were analyzed at different gonadal stages using qRT-PCR. In males, *kiss1* and *kiss2* exhibited maximal expression levels between the immature and early spermiation periods and gradually decreased in the post-spawning period (33). In females, *kiss2* expression reached a maximal level at the start of the vitellogenic period, and two receptors also showed significantly high expression at this time (Figure 4) (33, 36). Our previous study demonstrated that the gene expression of Gnrh1 was closely related to seasonal ovarian development, and Gnrh1 peptide secretion increased at the start of gonadal recrudescence (25). Collectively, these findings suggest that the activation of kisspeptin systems during gonadal recrudescence may influence Gnrh1 release from the brain to the pituitary.

Similar studies are abundant. A study of the seasonal expression of kisspeptin genes during the seasonal gonadal cycle in the adult grass puffer (*Takifugu niphobles*) found that the *kiss2* and *kissr2* genes in the brain were significantly elevated during the prespawning and spawning periods in both sexes (74). The *kiss1, kiss2*, and *kissr2* expression levels in the whole brain in male European sea bass were significantly higher in mid- and late spermatogenesis compared with the post-spawn period (75). Alvarado et al. measured the expression of kisspeptin-related genes in the hypothalamus of adult male and female European sea bass and reported that there genes increased either before or during the advanced stage of oogenesis and decreased during the atretic stage (76). In the rohu (*Labeo rohita*), the brain *kiss1* expression levels were significantly elevated at the prespawning and spawning periods in males and females, respectively (77). Similarly, in golden mahseer (*Tor putitora*), brain *kiss1* and *kissr3* expression levels were comparatively higher during the initial stages of gonadal development than during spermiation or ovulation (78). In the sapphire devil (*Chrysiptera cyanea*), brain *kissr2* and *kissr3* levels increased during the late vitellogenic and post-spawning periods (79).

Overall, kisspeptin system genes were activated just before or during the advanced stage of gonadal growth in many fish species. These observations suggest that the kisspeptin system is important for reproduction but may also be involved in various functions other than reproduction.

### Spawning Cycle

In the same manner, kisspeptin systems were found to be important during the spawning period in cm. We analyzed expression changes of kisspeptin-related genes in the brain during final oocyte maturation (FOM) and ovulation. Both *kiss1* and *kiss2* expression peaked during the FOM and ovulation stages (Figure 4) (80). Notably, the levels of Gnrh1 peptides also coincided with an increase in kisspeptin expression in the brain, and pituitary Lhb immunoreactivity was consistently high during FOM in the cm (27, 80). In contrast to other reproductive factors, the levels of kisspeptin receptors decreased during the FOM and ovulation phases (Figure 4) (81). In monkeys, continuous administration of human kisspeptin led to the desensitization of its receptor, Kiss1r (82, 83). Thus, desensitization of the kisspeptin receptors may have been involved in the decreased kisspeptin expression observed during the FOM and ovulation phases in cm.

Similarly, during their breeding seasons, medaka and goldfish displayed higher numbers of neurons expressing *kiss1* and *kiss2*, respectively, than they did during their non-breeding seasons (84, 85). In the grass puffer, both *kiss2* and *kissr2* showed clear diurnal and circadian variations in expression levels during the spawning season (86). In zebrafish, only males and females sampled at the time of spawning displayed strong *kiss2* expression in the periventricular hypothalamus (55).

In terms of breeding success, the estrogen feedback mechanism deserves a passing mention. Our previous study demonstrated that serum levels of E2 and its precursor, testosterone, showed characteristic variations during FOM and were significantly elevated when the largest and second largest oocytes were at the germinal vesicle migration stage and middle vitellogenesis stage of development, respectively (32). Interestingly, *kiss1* and *kiss2* expression levels peaked during FOM in the spawning cycle (80). Interactions between kisspeptins and steroid hormones require further investigation in cm. As in mammals, kisspeptin neurons express estrogen receptors and exhibit steroid sensitivity in medaka (84, 87), zebrafish (88), goldfish (85), and European sea bass (89).

## Pituitary Kisspeptin System

To provide a basic understanding of the involvement of kisspeptins in reproduction, we analyzed their gene expression in the pituitary at different gonadal stages using qRT-PCR. In cm, only *kiss1* and *kissr2* were expressed in the pituitary and did not show significant fluctuations during the annual reproductive cycle (33, 36). These results revealed that Kiss1 peptides exhibit lower affinity for KissR2 (36). The interactions between Kiss1 and KissR2 in the pituitary of cm remain unclear.

Functional analyses of the pituitary kisspeptin system have been reported in several species. *In vitro* studies on the actions of kisspeptin peptides have yielded conflicting results about their stimulatory effects on Lh release in goldfish (42, 90, 91). However, kisspeptin receptor expression was detected in immuno-identified gonadotrophs in the same species (90). An inhibitory effect of kisspeptin peptides on Lh secretion directly at the pituitary level was demonstrated in the European eel (92). In this species, *kissr1* and *kissr2* transcript levels were significantly downregulated in mature eels compared with in eels blocked at the prepubertal stage (11). In contrast, Kiss2-12, but not Kiss1-15, induced Fsh and Lh release from European sea bass pituitary cells, and Kiss2 cells also co-localized with gonadotropin-immunoreactive cells (93). In this species, *kiss2* and the two receptors increased either before or during the advanced stages of oogenesis in females (76). In a different study, in double transgenic *kiss2:mCherry*/*gnrh3:EGFP* zebrafish, both Kiss2 and Gnrh3 fibers extended to the pituitary *via* the pituitary stalk and were in direct contact with Gnrh3 fibers in the pars distalis (94). In addition, Zmora et al. reported that the Kiss1 and Kiss2 nucleus lateralis tuberis (NLT) populations probably act directly on pituitary gonadotrophs at the prespawning stage in male and female striped bass (55). The same study also reported that both Kiss1-15 and Kiss2-12 induced Fsh release, and Kiss2-12 induced Lh release from pituitary cells *in vitro*. The role of the pituitary kisspeptin system is still unknown. However, this system may be important in gonadotropin regulation in several teleost species, as these actions are independent of Gnrh signaling. More detailed studies should be performed in the near future to further clarify the role of the pituitary kisspeptin system in cm.

## Gonadal Kisspeptin System

It should also be noted that the kisspeptin system has an important role at the gonadal level. Our qRT-PCR analysis showed that *kiss1* and *kissr3* (cognate receptor of Kiss1) were expressed strongly in the testes and dramatically increased at the spermiation stage of the annual reproductive cycle in adult cm (33, 36). In particular, the *kissr3* transcript level was 10.8-fold higher at this stage than at the immature and post-spawning periods (36). The possibility that there are autocrine/paracrine effects of Kiss1 peptides on the testes cannot be excluded, but our expression and pharmacological analyses suggest a local role of the Kiss1 system in the testes of cm. Notably, ovarian *kiss1* expression significantly increased during vitellogenesis, but the ovaries did not express any kisspeptin receptors (33, 36). One possible explanation is that ovarian kisspeptin may exert effects *via* other RFamide receptors in this species. As a simple example, kisspeptins mediate physiological effects *via* the neuropeptide FF1 and FF2 receptors in mammals (95, 96).

At the gonadal level, a positive correlation between gonadal development and kisspeptin system expression has been reported in several species. In the grey mullet, ovarian *kissr2* expression showed an increasing trend during early development (64). In European sea bass, testicular *kissr2* expression exhibited a significant increase at the beginning of spermiation, and *kissr3* levels increased significantly in the full spermiation stage (37). In rohu, gonadal *kiss1* expression increased during the prespawning and spawning periods in male and female fish, respectively (77). In male golden mahseer, consistently high transcript levels of *kiss1* were observed during testicular development and, in female fish, *kiss1* and *kissr2* expression peaked in the late vitellogenic ovary (78). In male pejerrey, *kiss1* and *kissr3* increased during testicular development compared with their levels in immature testes, and female fish showed high levels of expression of receptors in the ovaries at final maturation (97).

The significance of kisspeptin expression in the fish gonad is still unknown but is a promising area of future research not only in cm but also in other fish species.

## Anatomy

### Localization of Kisspeptin Neurons

The lack of anatomical evidence for a neuronal network is the central problem in elucidating the functions of kisspeptin in fish. We analyzed the localization of two kisspeptin neurons in the brains of adult cm using *in situ* hybridization (ISH). *Kiss1* mRNAs were detected in the anterior part of the POA, the ventral hypothalamus, including the NLT, and the dorsal hypothalamus, including the nucleus recessus lateralis (38). *Kiss2* mRNAs were detected in the anterior POA and NLT, and a large population was observed in the ventral hypothalamus, including the NRL (38). In our experiment, no sex differences were observed. The localization of neurons expressing the two kisspeptins has been reported in detail in five fish species: medaka (47, 84), zebrafish (88), striped bass (17), European sea bass (23, 89), and goldfish (85, 98). Furthermore, histological *kiss2* expression was examined in four species: red sea bream (73), Nile tilapia (99), masu salmon (39), and grass puffer (86). The distribution of cells expressing cm *kiss1* and *kiss2* roughly matched those of the four species mentioned above.

In fish, highly specific Kiss1 and Kiss2 antibodies were developed in zebrafish (88), and a Kiss2 antibody was developed in European sea bass (23). Immunohistochemistry (IHC) revealed that Kiss2 neurons are mainly located in the hypothalamus and project widely to the subpallium, the POA, the thalamus, the ventral and caudal hypothalamus, and the mesencephalon in zebrafish and European sea bass (23, 88). All these regions strongly expressed the *kissr2* mRNAs (see next section), indicating a very strong correlation with the wide distribution of Kiss2-positive fibers. On the other hand, a large population of Kiss2 neurons was localized in the POA region in goldfish and showed a strong sensitivity for estrogen feedback (85). This suggests that POA Kiss2 neurons play an important role in the regulation of reproduction in this species.

Kiss1 localization also varies among species. In zebrafish, medaka, and European sea bass, the main location of Kiss1 neurons and receptors is the habenular nucleus, and they may function by autocrine or paracrine action. Indeed, double-labeled ISH and *c-fos* expression after Kiss1 administration suggested the existence of autocrine regulation in zebrafish habenular nuclei (100). However, in our study, Kiss1 neurons were not expressed in the habenular nucleus, and the same result was reported in striped bass (17, 38). The physiological function or significance of the fish Kiss1 system remains largely unknown. Studies in goldfish found a dominant role of Kiss1 in the regulation of Lh secretion *in vivo* and *in vitro* (42, 90, 91). On the other hand, habenular Kiss1 modulates the serotonergic system and fear response in zebrafish (100, 101). In IHC studies in zebrafish, Kiss1 immunoreactive neurons were found in the ventromedial habenula, with axons contacting the interpeduncular and raphe nuclei, which express serotonergic neurons (88, 100). Clarifying the Kiss1 system in fish is an important and interesting future task; however, this peptide likely contributes to reproduction in cm and will be described later.

### Localization of Kisspeptin Receptors

We also examined the localization of *kissr2*- and *kissr3*-expressing cells in the brains of cm using ISH. The *kissr2*- (cognate receptor of the Kiss2 peptide) expressing cells displayed a broader distribution in the anterior and posterior parts of the POA and were abundant in the ventral hypothalamus, including the ventral and lateral parts of the NLT; the ventral, lateral, and dorsal parts of the NRL; and the nucleus of the NRP (38). The *kissr3*- (cognate receptor of the Kiss1 peptide) expressing cells were present in the anterior POA and the dorsal and ventral parts of the habenular nucleus (38). No expression differences between the sexes were observed.

The localization of the two types of kisspeptin receptor-expressing cells has been reported in detail in zebrafish (88), striped bass (17), European sea bass (23), medaka (22), and in one species of African cichlid fish with only the *kissr2* transcript (16). The distributions of cells expressing kisspeptin receptors roughly matched and, overall, *kissr2* showed widespread, high expression levels, whereas *kissr3* was modestly expressed in limited regions of the brain.

### Interactions With Functional Gnrh-Producing Cells

**Figure 5:**
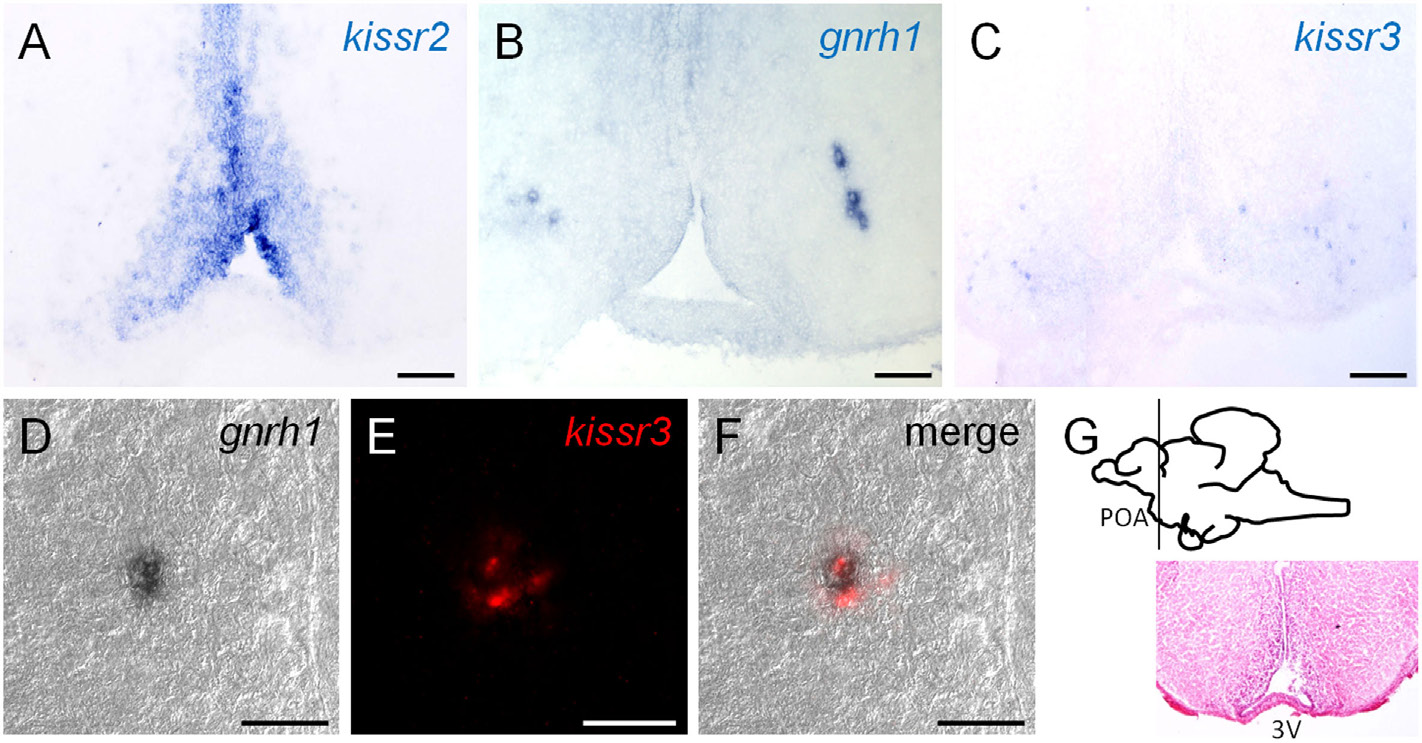
**(A–C)** Localization of *kissr2* expressing cells, Gnrh1 neurons, and *kissr3*-expressing cells at pre-optic area (POA), respectively. Scale bars, 100 µm. **(D–F)** Double-label *in situ* hybridization at the POA region. Scale bars, 30 µm. **(D)** Differential interference contrast images of Gnrh1 neurons. **(E)** Red fluorescence indicate the *kissr3* mRNA. **(C)** Merged image of **(E,F)** and indicate that Gnrh1 neurons co-expressed *kissr3* mRNA. **(G)** Sagittal view of the chub mackerel brain. Planes of the ventral POA region are visualized by hematoxylin–eosin staining. Modified from Ref. (38), by permission of Oxford University Press.

Central to the fish kisspeptin issue is the problem of interactions with Gnrh neurons. To investigate this, we conducted dual-labeled ISH and carefully assessed the signals using confocal laser-scanning microscopy. Our study clarified that Gnrh1 neurons co-expressed *kissr3* mRNA in the POA region in male and female cm (Figures 5A–G) (38). There is no doubt that the Kiss1 peptide is involved in reproduction, and some of its signals are inputs for Gnrh1 neurons. As noted earlier, Kiss1 treatment strongly induced gonadal development in cm (48, 50). In addition, *kissr2* was widely expressed in the POA; in many cases, it was expressed in close proximity to the Gnrh1 neurons, but they did not appear to be co-expressed (38).

The interactions between kisspeptin receptors and functional Gnrh neurons were first examined in a cichlid fish, Nile tilapia, using laser-captured single-cell PCR (10). The results clearly showed that all three types of Gnrh neuronal cells (Gnrh1, 2, and 3) expressed *kissr2* mRNA (10). This observation was supported by double-labeled ISH analysis by Grone et al., which showed that Gnrh1 and Gnrh3 neurons were co-expressed with *kissr2* transcripts in cichlid fish (16). Furthermore, *in vivo* treatment with Kiss2 modulated the reproductive axis and gonadal development in the same species, supporting the histological evidence (54). The same anatomical evidence was reported in a study in striped bass combining ISH and IHC, which showed that Gnrh1 cell bodies co-expressed *kissr2* mRNA, and *kissr3* was expressed in cells attached to Gnrh1 fibers (17). This may suggest dual modes of Gnrh1 regulation by the two kisspeptin peptides; indeed, both Kiss1 and Kiss2 enhanced Lh secretion in adult fish (17).

Alternatively, Gnrh neurons lack the expression of kisspeptin receptors in some species. An IHC study in zebrafish reported that some Gnrh3 neurons were contacted by Kiss2 fibers (88). Similarly, in double transgenic *kiss2:mCherry*/*gnrh3:EGFP* zebrafish, Kiss2 and Gnrh3 fibers were adjacent, and few contacts were observed in the telencephalon and hypothalamus (94). However, both studies similarly failed to find evidence of kisspeptin receptors in Gnrh3 neurons. In addition, morphological evidence from dual-labeled fluorescence ISH has shown that Gnrh1 neurons do not appear to express *kissr2* or *kissr3* mRNAs in medaka (22). In European sea bass, an antibody against preproGnrh1 was used for coupling with ISH for kisspeptin receptors. In this study, both *kissr2*- and *kissr3*-expressing cells in the ventral telencephalon and POA were often found in close proximity to Gnrh1 neurons; however, the authors did not detect a single case of co-expression (23). Nevertheless, administration of Kiss2 peptides upregulated the expression of the gonadotropin subunit genes as well as the secretion of gonadotropins in zebrafish and European sea bass, respectively (18, 19, 47). Based on the numerous studies of fish kisspeptins, it seems reasonable to suggest that kisspeptins affect gonadotropin regulation. However, the pathway of gonadotropin regulation may vary among species. The relationship between kisspeptin and Gnrh alone may not form the core of kisspeptin function in fish reproductive physiology.

### Interactions With Other Factors

Finally, there is one other important factor for kisspeptin function in fish. Despite the lack of direct contact between Kiss2 and Gnrh1 neurons, i.c.v. administration of Kiss2 peptides strongly enhanced the transcription of gonadotropins and influenced *gnrh1* expression in cm (45). Our histological observations showed that *kissr2* was abundantly expressed in the vicinity of Gnrh1 neurons and hypothalamic regions (38). The transcript levels were quantified by qRT-PCR, and *kissr2* transcripts were expressed at far higher levels than were *kissr3* transcripts, indicating that the expression of *kiss2*/*kissr2* can be very dynamic, depending on maturational stage, as shown in many previous studies in cm (33, 36). One hypothesis is that Kiss2 indirectly regulates the reproductive axis *via* interneurons that are expressed in close proximity to Gnrh1 neurons.

In the earliest study, Grone et al. discovered that not only Gnrh1 neurons but also some unknown non-Gnrh cells in the vicinity of Gnrh1 neurons expressed *kissr2* mRNA in African cichlid fish (16). Recent studies have clarified that various types of neurons co-express kisspeptin receptors in fish. Escobar et al. reported that neuronal nitric oxide synthase (nNOS), neuropeptide Y (NPY), tyrosine hydroxylase, and somatostatin (SRIF) neurons co-expressed *kissr2* transcripts in the brains of European sea bass (23). In mice, Gnrh neurons are surrounded by nNOS neurons, which express *Kiss1r*, and nNOS may contribute to the direct modulation of Gnrh neuronal activity in a manner codependent with kisspeptin signaling (102, 103). Also, NPY was shown to induce Lh secretion in European sea bass (104). Gnrh1 neurons in European sea bass do not express *kissr2* mRNA; it is likely that Kiss2 regulates Gnrh and gonadotropin secretion *via* various neuronal networks.

Kisspeptin may also play an important role in reproductive behavior. Grone et al. reported that significantly more *kissr2* transcripts were found in Gnrh3 neurons than in Gnrh1 neurons in African cichlid fish (16). The same report also clarified that high-status territorial males have higher brain levels of *kissr2* mRNA than low-status non-territorial males. Some teleost fishes have three distinct populations of Gnrh neurons, and Gnrh3 neurons are considered to be particularly important for reproductive behaviors (105). On the other hand, Zhao and Wayne reported changes in Gnrh3 electrical activity after the application of Kiss1 through some interneurons in medaka (106). Furthermore, breeding medaka showed changes in hypothalamic *kiss1* and telencephalic *gnrh3* expression depending on differences in dominance hierarchy (107). In the same species, Kanda et al. showed that arginine vasotocin (Avt) and isotocin (It) neurons co-expressed *kissr2* mRNA in the brains (22). In teleost fish, Avt and It are mainly implicated in spawning reflex, courtship and mate-guarding behavior, and hierarchical status (108, 109). These results suggest that kisspeptin neurons directly regulate some sexual behavioral functions *via* Gnrh3 neurons or Avt and It neurons. The correlation between kisspeptin systems and behavioral factors were similarly reported in striped bass: Avt neurons expressed *kissr2* mRNA, and It neurons expressed *kissr3* mRNA (20).

In another case, pituitary growth hormone (Gh)-producing cells expressed *kiss1* and its receptor transcripts, and pituitary prolactin-producing cells also expressed *kissr2* mRNA in goldfish (90). In the same study, Kiss1-10 increased the basal release of Gh and prolactin from pituitary cells *in vitro*. Furthermore, administration of human KP10 promoted Gh, insulin-like growth factor 1 (IGF-I), and somatolactin secretion and pituitary gene expression in cinnamon clownfish (110). As mentioned earlier, in European sea bass, which is the same marine Perciform species, SRIF neurons co-expressed kisspeptin receptors (23). SRIF is a highly conserved peptide that also acts as an inhibitor of Gh secretion in teleosts (111, 112). Although seemingly contradictory, these results point to the potential function of kisspeptins in regulating somatic growth-related factors.

The kisspeptin/neurokinin B/dynorphin A (KNDy) neurons will play a key role in regulating pulsatile secretion of Gnrh in mammals (113), but very little is known regarding the relationship between neurokinins and kisspeptins in the context of reproduction in fish. In past study, i.p. administration of tachykinins cording peptides, neurokinin B (NKB) and neurokinin F (NKF: unique neurokinin form in fish) elicited significant Lh secretion in sexually mature female zebrafish (114). However, ISH showed no co-expression of tachykinins mRNA with kisspeptins mRNA (115). On the other hand, i.m. administration of NKB and NKF reduced *kiss1* and *kiss2* gene expression in the brain and pituitary content of Gnrh1 in spermiating striped bass (116). Furthermore, tachykinin (*tac3*) neurons in the hypothalamus strongly innervated proximal Kiss2 neurons in the dorsal and ventral NRL, which in turn express cognate receptor (*tac3r*) (116). Finally, we may note in passing that a recent deep-sequencing study suggested that novel neural systems, such as cholecystokinin and neuropeptide B, may also be under the control of kisspeptin signals in medaka (117). The multiple and integral regulation of the reproductive axis or non-reproductive functions by fish kisspeptins are still largely unknown and in need of further consideration.

## Perspectives

In this article, we reviewed the current insights on fish kisspeptin physiology based on our cm studies. A take-home message from this article is that the kisspeptin system plays a role in the reproductive success not only of cm but also of many other fish species *via* various known or unknown neuronal networks. It must be noted that the methods of involvement differ from species to species. The correlation between kisspeptins and Gnrh neurons is a good illustration of the high species specificity of the kisspeptin system. Namely, it may be erroneous to assume that fish kisspeptin is a central and absolute upstream regulator of the Gnrh–Gth pathway as is the case in mammals. Fish are known as the earliest vertebrates, and it may be that their hierarchical reproductive fine network has not yet been completed.

A recent genome editing study clearly showed that kisspeptin-related genes null mutant zebrafish or medaka showed normal gonadal development and maturation (21, 117). However, in fish, various neuropeptidergic, catecholaminergic, and amino-acidergic neurons form direct or indirect contacts with gonadotrophs in the pituitary gland, possibly constituting a multiple back-up system to maintain appropriate gonadotropin release. This does not affect the validity of the reproductive functions of fish kisspeptins.

The strong potency of gonadotropin release is relevant to our research objectives. With capture fishery production relatively static since the late 1980s, aquaculture has been responsible for the impressive growth in the supply of fish for human consumption (118). Hence, the development of efficient aquaculture methods and a model of reproduction are imperative tasks. The “maturation induction” potency of kisspeptin peptides may have the potential to rescue reproductive failure in cultured fish. Additional comparative kisspeptin research will further elucidate the pleiotropic role of kisspeptin in fish and may contribute to the development of not only a basic understanding of fish physiology but also of the applied science used in aquaculture and stock management.

## Author Contributions

HO wrote a manuscript. HO, SS, and MM contributed substantially to the conception and design of the work, reviewing, final approvals of the version submitted, and agreed to be accountable for accuracy and integrity of content.

## Conflict of Interest Statement

The authors declare that the research was conducted in the absence of any commercial or financial relationships that could be construed as a potential conflict of interest.
